# Skin-Microbiome Assembly in Preterm Infants during the First Three Weeks of Life and Impact of Topical Coconut Oil Application

**DOI:** 10.3390/ijms242316626

**Published:** 2023-11-22

**Authors:** Noor-Ul-Huda Ghori, Christopher A. Mullally, Mark P. Nicol, Andrew Currie, Julie Hibbert, Matthew S. Payne, Sanjay Patole, Tobias Strunk

**Affiliations:** 1 Division of Infection and Immunity, School of Biomedical Sciences and The Marshall Centre, The University of Western Australia, Perth 6009, Australiamark.nicol@uwa.edu.au (M.P.N.); 2 Wesfarmers Centre for Vaccines and Infectious Diseases, Telethon Kids Institute, Perth 6009, Australia; 3 Centre of Molecular Medicine and Innovative Therapeutics, Murdoch University, Perth 6150, Australia; 4 Division of Obstetrics and Gynecology, School of Medicine, The University of Western Australia, Perth 6009, Australia; 5 Neonatal Directorate, King Edward Memorial Hospital for Women, Child and Adolescent Health Service, Perth 6008, Australia; 6 Faculty of Health and Medical Sciences, The University of Western Australia, Perth 6009, Australia

**Keywords:** coconut oil, preterm infants, skin microbiome

## Abstract

The structure and function of infant skin is not fully developed until 34 weeks of gestation, and this immaturity is associated with risk of late-onset sepsis (LOS). Topical coconut oil improves preterm-infant skin integrity and may reduce LOS. However, data on early-life skin-microbiome succession and potential effects of emollient skin care in preterm infants are scarce. We therefore collected skin-microbiome samples from the ear, axilla, and groin on days 1, 7, 14, and 21 from preterm infants born <30 weeks of gestation as part of a randomized clinical trial of standard skin care vs. topical coconut oil. We found that within-sample microbiome diversity was highest on day 1 after birth, with a subsequent decline and emergence of *Staphylococcus* genus dominance from day 7. Moreover, microbiome assembly was less diverse in infants receiving coconut oil vs. standard skin care. Our study provides novel data on preterm-infant skin-microbiome composition and highlights the modifying potential of emollient skin care.

## 1. Introduction

Human skin hosts a diverse, low-density microbiome of bacteria and fungi [1]. Emerging evidence suggests that commensal skin microbiota plays a fundamental role in regulating the physical integrity and repair of the skin barrier [2] and is crucial for developing the infant immune system [3,4]. The skin microbiota stimulates the development of skin immune cells and the production of antimicrobial peptides, short-chain fatty acids, and polyamines, which are critical for defense against infection [5]. The commensal skin microbiota produce metabolites that activate the aryl hydrocarbon receptor (AHR) in keratinocytes [2], which, in turn, accelerates epidermal differentiation and increases stratum corneum thickness. Moreover, commensal skin microbiota, such as *Staphylococcus epidermidis*, enhance skin barrier function by stimulating keratinocytes to produce ceramides, which are essential in reducing trans-epidermal water loss (TEWL) [6]. 

Skin colonisation begins immediately at, or even before, birth; however, the factors influencing the succession of the skin microbiota are not completely understood. For example, the effect of the mode of delivery on skin-microbiome composition remains controversial [7,8,9,10]. The skin of extremely-to-moderately preterm infants does not provide an adequate protective barrier against infection and water loss. The stratum corneum, the external epidermal layer of the skin, begins to develop at 24 weeks of gestation. This layer has several cornified layers at 26 weeks and is fully developed by 34 weeks of gestation [11]. A mature stratum corneum provides a critical physical barrier, prevents invasion by microorganisms, and minimizes TEWL, preventing dehydration [11]. 

Infant skin at birth is initially rich in Firmicutes, followed by Actinobacteria, Proteobacteria, and *Bacteroides*. Preterm infants may have a higher skin abundance of Firmicutes, including *Staphylococcus*, *Corynebacterium*, and *Prevotella* bacteria, than full-term infants [8]. Since preterm infants are frequently exposed to antibiotic therapy and parenteral nutrition and are hospitalized for prolonged periods, they are susceptible to nosocomial infections, commonly with skin commensals [8]. Coagulase-negative staphylococci (CoNS), including *S. epidermidis* and *S. capitis*, colonize preterm infants and account for >75% of cases of late-onset sepsis (LOS) [12,13]. The vast majority of very preterm infants are exposed to empiric antibiotic therapy during their hospital stay, often repeatedly, and this may decrease cutaneous bacterial diversity [14].

Emollients used for neonatal skin care, both mineral-oil-derived and natural oils, aim to reduce TEWL and improve skin integrity [15], but their effects on skin barrier function and LOS in preterm infants are not fully understood. Skincare emollients may also have inadvertent deleterious effects on the barrier function of the skin of neonates, such as impairment of epidermal maturation by oleic acid in olive oil [16] or increase in sepsis risk in the smallest infants following the use of Aquaphor [17]. Coconut oil may be a more suitable emollient for neonatal skin care due to its high concentrations of lauric acid and its ester monolaurin, both with antimicrobial properties [18,19], and the absence of known adverse effects [20]. However, its effects on the neonatal skin microbiota have not been investigated. 

Our previously described, randomized, controlled trial of topical coconut oil in very (born <30 weeks GA) preterm infants resulted in improved skin integrity, without adverse effects [19]. Here, we describe skin-microbial community assembly and the effect of the coconut oil intervention on the skin-microbial community in very preterm infants enrolled in this trial during the first 3 weeks of life. 

## 2. Results

We collected skin swabs from 72 very preterm infants from the axilla, ear, and groin on days 1, 7, 14, and 21 after birth. Microbiome analysis was performed on samples with sufficient DNA content from 672 swab samples, from a total of 56 infants (28 infants each in intervention and control arms, respectively). The demographics of participants are described in Table 1. During the study intervention, there was one episode of LOS in the coconut oil intervention arm and three episodes in the control arm.

For skin-microbiome analysis, a total of 52,972,312 reads were obtained from 672 swab samples. A total of 14,912 ASVs were assigned to reads after denoising with the DADA2 pipeline. The ASVs were assigned to 34 phyla, 81 classes, 209 orders, 354 families, 819 genera, and 540 species. We identified 40 ASVs as potential contaminants, using the Decontam pipeline [21] (Supplementary Table S1), and removed these ASVs from the feature table.

### 2.1. Succession of the Skin Microbiota of Preterm Infants in Both Control and Intervention Infants

**Figure 1:**
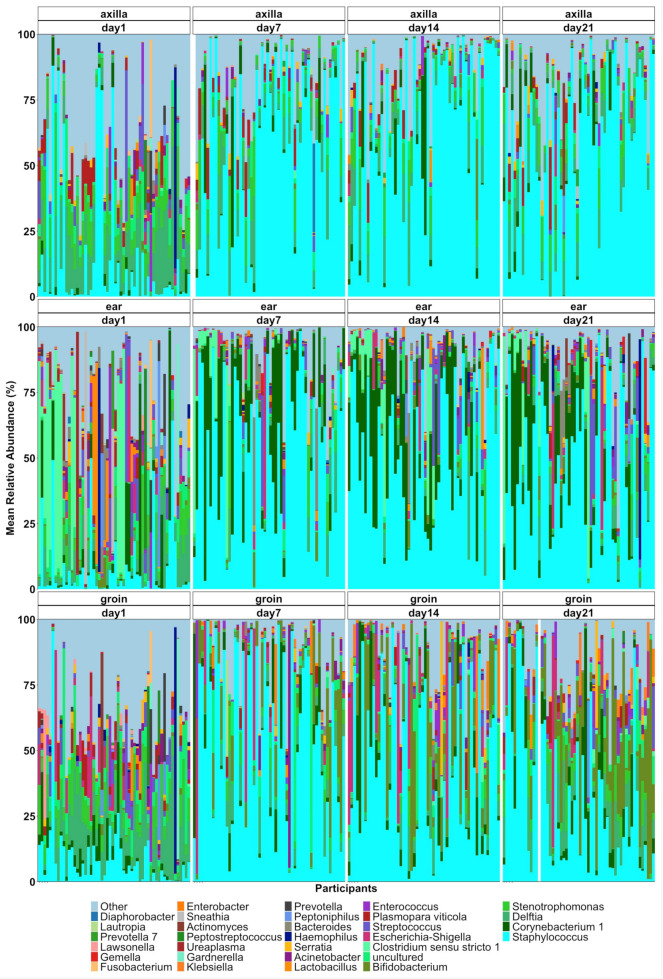
Relative abundance of bacterial taxa in individual participants. Compositional bar plot shows the mean relative abundance at the genus level for each individual participant at axilla, ear, and groin sites for samples collected on days 1, 7, 14, and 21. Genera that had a relative abundance of <10% are listed as “other”.

There were substantial changes in the composition of the skin microbiome over the first three weeks of life (Figure 1). These were further visualized for age at sampling as longitudinal data using an alluvial plot for each body site (Supplementary Figure S1). At all sites, the *Staphylococcus* genus increased in relative abundance with age at sampling, most strikingly in the axilla. The groin skin microbiome was characterized by a profile dominated by the *Burkholderia* or *Staphylococcus* genus on day 1 but was dominated by *Staphylococcus* or *Bifidobacterium* at ages 7, 14, and 21 days. In differential abundance analysis, *Staphylococcus* showed higher relative abundance on day 7 (*q*-value = 2.43 × 10^−9^ and day 14 (*q*-value = 2.18 × 10^−9^) compared to day 1 samples (Table 2). *Bifidobacterium* (*q*-value = 0.006) and *Cutibacterium* (*q*-value = 0.0003) also showed increased relative abundances on day 7 and day 14 compared to day 1. *Clostridium* (*q*-value = 9.46 × 10^−5^) had higher relative abundance in the axilla vs. groin samples, while the opposite finding was observed for *Streptococcus* (*q*-value = 0.002).

**Figure 2:**
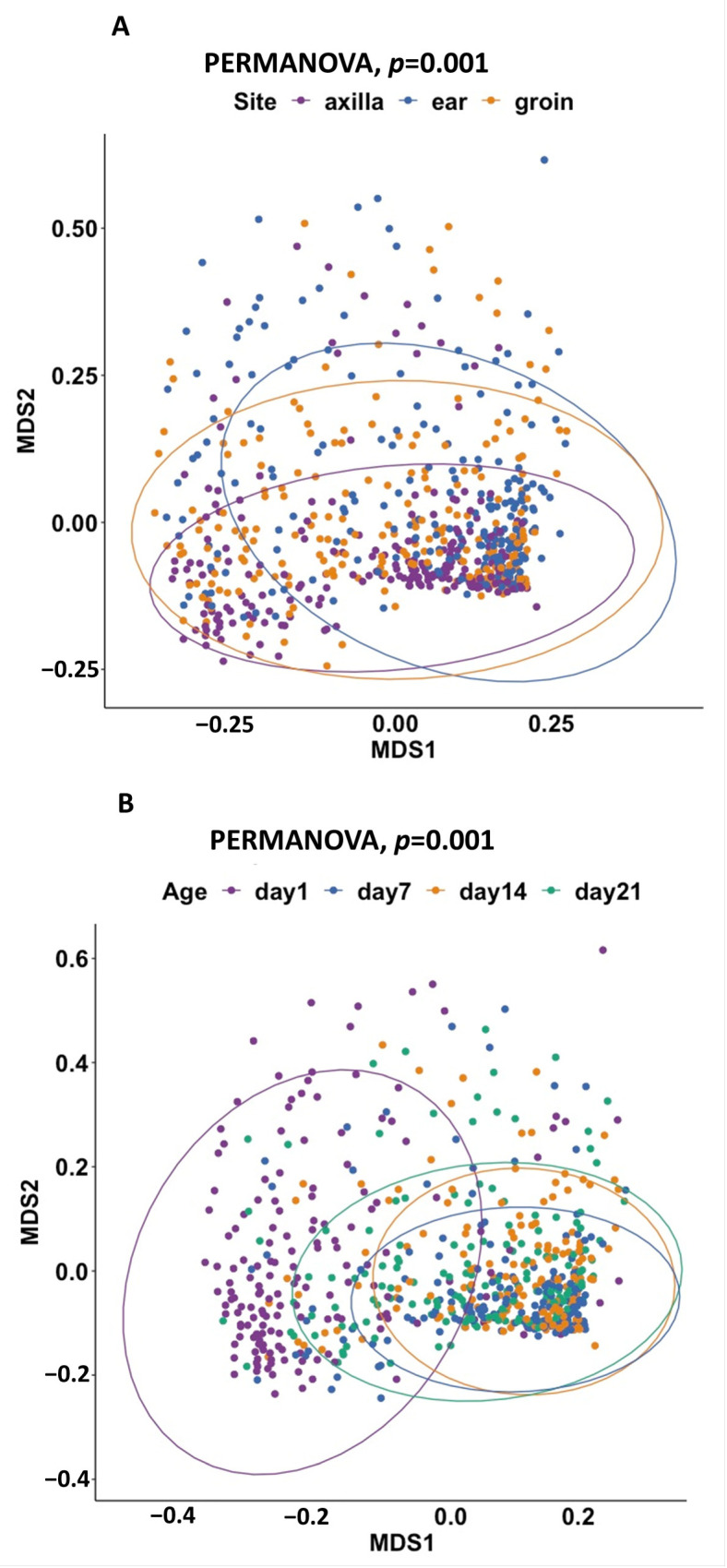
(**A**) Beta-diversity NMDS plot of different body sites. Non-metric-multidimensional-scaling (NMDS) plot at ASV level using Bray–Curtis dissimilarity index (PERMANOVA, *p*-value = 0.001). (**B**) Beta-diversity NMDS plot of age at sampling. Non-metric-multidimensional-scaling (NMDS) plot at ASV level using Bray–Curtis dissimilarity index (PERMANOVA, *p*-value = 0.001).

The dissimilarity between microbial communities at different body sites and ages at sampling was assessed with non-metric multidimensional scaling (NMDS) using the Bray–Curtis dissimilarity index. Between-body-site distances were greater than within-site distances (PERMANOVA, *p* = 0.001) (Figure 2A). β-diversity also differed with age at sampling, with day 1 samples clearly separated from samples collected on later days (PERMANOVA, *p* = 0.001) (Figure 2B). 

**Figure 3:**
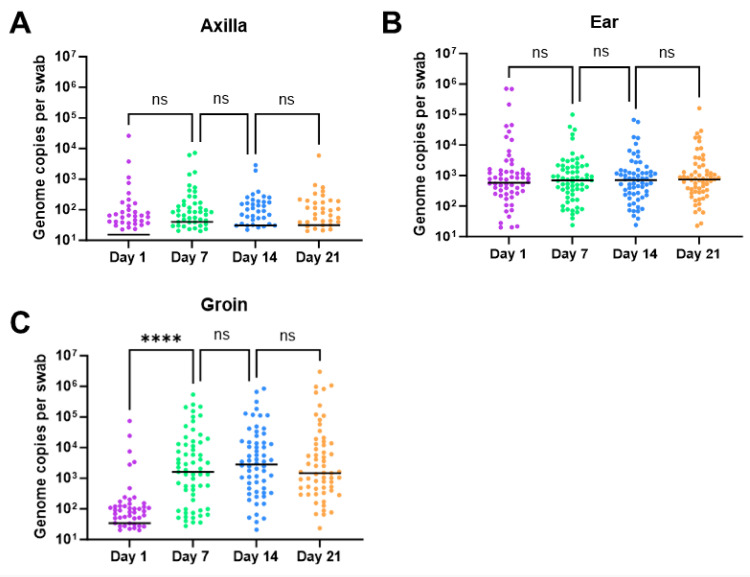
Quantitative PCR data for *Bifidobacterium* spp. at all time points for (**A**) axilla, (**B**) ear, and (**C**) groin. DNA in ng was measured using a standard curve and converted to total genomes per swab. Zero values are plotted as half the limit of detection (8 genome copies per swab). The median value for each time point is represented by a black bar. Each time point was compared with the other using a Kruskal–Wallis analysis of variance with Dunn’s multiple comparisons test. Comparisons with significant differences are denoted in each figure (**** = *p* < 0.0001 and ns = *p* > 0.05). Each time point is represented by a different color (day 1 = purple, day 7 = green, day 14 = blue, and day 21 = orange).

Using qPCR for *Bifidobacterium* spp., the proportion of swabs that were positive for the groin, ear, and axilla were 82.1%, 59.0%, and 95.4%, respectively. However, the axilla had the lowest *Bifidobacterium* bacterial load, the ear had an intermediate *Bifidobacterium* load, and the groin swabs had the highest load, with an increase from day 1 to day 7 (*p* ≤ 0.0001) (Figure 3). 

### 2.2. Factors Associated with Diversity of the Preterm-Infant Skin Microbiome

**Figure 4:**
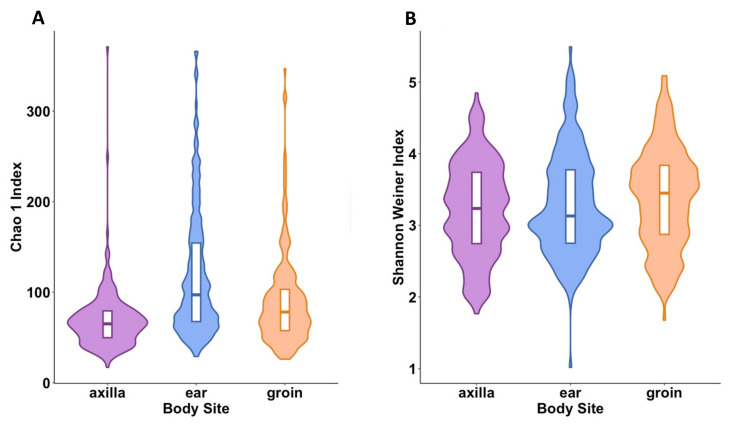
Alpha-diversity metrics for body site: axilla, ear, and groin. (**A**) Violin plot of Chao1 index (axilla: *p*-value ≤ 0.001 and β = −28.07; ear: *p*-value ≤ 0.001 and β = 28.07; both vs. groin). (**B**) Violin plot of Shannon–Weiner index (axilla: *p*-value = 0.006 and β = 0.16; ear: *p*-value = 0.11 and β = −0.09; both compared with groin). The box plot represents the interquartile range, and the middle line represents the median. The colored shape of the violin plot represents the density of measurements.

We tested the association between various participant-level factors and alpha diversity (within sample diversity) using linear mixed models. Bacterial richness (Chao1) in axilla samples was lower than that in groin samples (*p* < 0.001), while ear samples had higher richness than groin samples (*p* < 0.001) (Figure 4A). Using a different metric for α-diversity (Shannon–Weiner index, which also accounts for the evenness of taxa within samples) (Figure 4B), groin samples had higher diversity compared with the axilla or ear samples.

**Figure 5:**
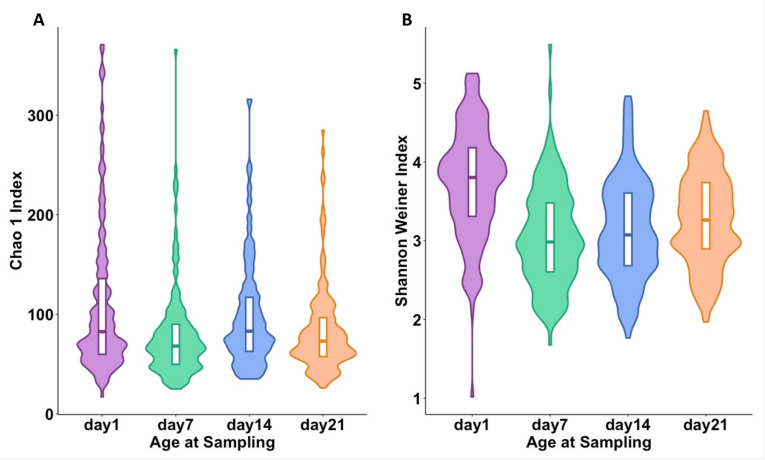
Alpha-diversity metrics for age at sampling: day 1, day 7, day 14, and day 21. (**A**) Violin plot of Chao1 index (day 7: *p*-value ≤ 0.001 and β = −30.7; day 14: *p*-value = 0.02 and β = −11.95; day 21: *p*-value ≤ 0.001 and β = −26.59; all compared to day 1). (**B**) Violin plot of Shannon–Wiener index (day 7: *p*-value ≤ 0.001 and β = −30.75; day 14, *p*-value = 0.02 and β = −11.95; day 21: *p*-value ≤ 0.001 and β = −26.58, all compared to day 1). The box plot represents the interquartile range, and the middle line represents the median. The colored shape of the violin plot represents the density of measurements. Each time point is represented by a different color (day 1 = purple, day 7 = green, day 14 = blue, and day 21 = orange).

Bacterial richness was lower on day 7 (*p* < 0.001), day 14 (*p* = 0.021), and day 21 (*p* < 0.001) compared to day 1 (Figure 5A). Similar findings were observed using the Shannon diversity index (Figure 5B). 

We also analyzed the association between the total duration of antibiotic therapy, prior to each sampling time point, and alpha diversity. Lower Shannon diversity index was similar to prior antibiotic treatment (*p* = 0.981). 

### 2.3. Effect of Topical Coconut Oil on the Skin Microbiome

**Figure 6:**
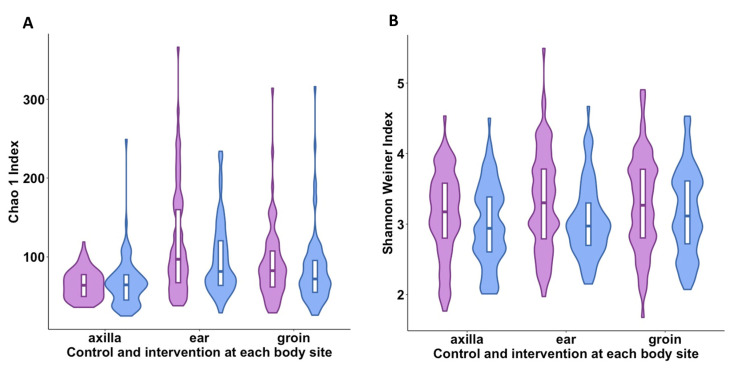
Alpha-diversity metrics for control and intervention arms at each body site. (**A**) Violin plot of Chao1 index (intervention: *p*-value = 0.12 and β = −9.97) and (**B**) violin plot of Shannon–Weiner index (intervention: *p*-value = 0.006 and β = −0.16) for control and intervention arms at axilla, ear, and groin body sites. The box plot represents the interquartile range, and the middle line represents the median. The colored shape of the violin plot represents the density of measurements.

**Figure 7:**
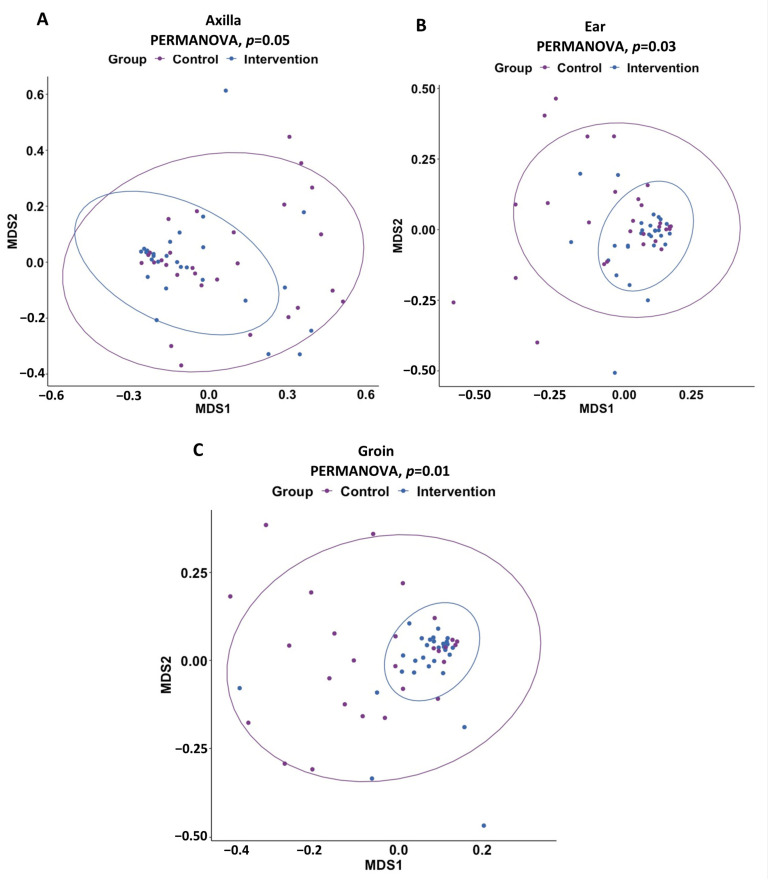
Beta-diversity NMDS plot for (**A**) axilla, (**B**), ear, and (**C**) groin in the intervention, and control arm on day 7. Beta diversity is shown in a non-metric-multidimensional-scaling (NMDS) plot using the Bray–Curtis dissimilarity index for each body site in control and intervention arms for day 7 samples.

We analyzed the effect of coconut oil emollient on within-sample diversity using a linear mixed model with participant ID as a random effect and with site and age as fixed effects. Compared to the control arm, there was a trend towards reduced microbiome richness in the intervention arm (beta = −9.97, 95% CI [−22.68, 2.74], *p* = 0.124). Using the Shannon–Weiner index of diversity, the intervention was negatively associated with diversity (beta = −0.16, 95% CI [−0.27, −0.04], *p* = 0.006) (Figure 6A,B). The overall composition of the skin microbiome, as measured by Bray–Curtis dissimilarity, differed between control and intervention arms on day 7 only for ear (PERMANOVA, *p* = 0.01) and groin (PERMANOVA, *p* = 0.02) samples (Figure 7). There were differences in individual taxa between the intervention and control arms using Maaslin2 analysis of the microbiome.

Using qPCR, we analyzed the number of genome copies of CoNS and *S. aureus* in all samples. None of the axilla samples had any detectable *S. aureus* (Supplementary Table S2). Only two participants had detectable levels of *S. aureus* on day 7, both in the intervention arm and both from the groin. On day 14, two participants had *S. aureus* detected, one from a groin swab and the other from an ear swab. Both of these participants were in the control arm. Finally, on day 21, four participants had detectable levels of *S. aureus*, all in the control arm, from groin swabs (Supplementary Table S2). Except for one participant, who was positive for *S. aureus* on days 14 and 21, *S. aureus* detection was transient.

**Figure 8:**
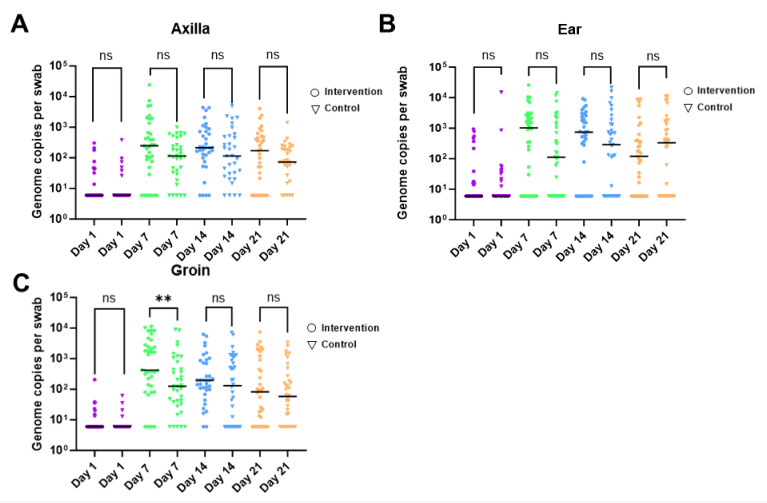
Quantitative PCR data for coagulase-negative staphylococci from participants at all time points for (**A**) axilla, (**B**) ear, and (**C**) groin. DNA in ng was measured using a standard curve and converted to total genomes per swab. Zero values are plotted as half the limit of detection (8 genome copies per swab). The median for each arm/time point is represented by a black bar. Each time point is represented by a different color (day 1 = purple, day 7 = green, day 14 = blue, and day 21 = orange). Each time point was compared between the treatment and control arms using a Mann–Whitney test. Comparisons with significant differences are denoted in each figure (** = *p* < 0.01, and ns = *p* > 0.05).

CoNS DNA was detected by qPCR in 66.8% of all samples. On day 1, CoNS DNA was detected in 40 participants (55.56%) from at least one site. By day 7, 70 participants (97.2%) had detectable levels of CoNS DNA from at least one site, with a concurrent increase in copy number. The CoNS load remained stable from day 7 onwards (Figure 8A).

**Figure 9:**
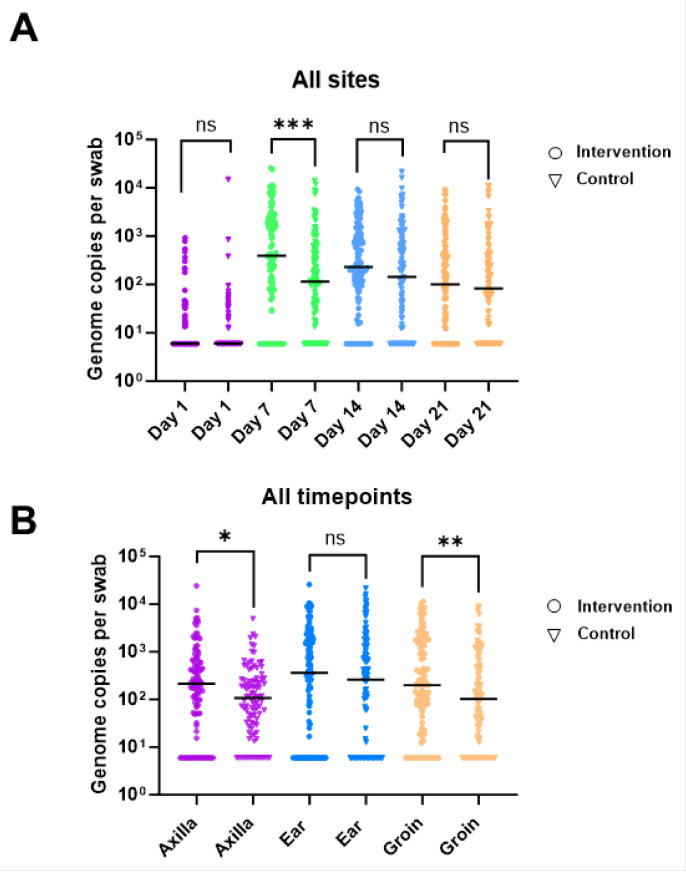
Quantitative PCR data for coagulase-negative staphylococci from participants with combined sites and time points. DNA in ng was measured using a standard curve and converted to total genomes per swab. The median for each arm/time point is represented by a black bar (when a median value is 0, the bar is not shown). Each time point was compared between the intervention and control arms using a Mann–Whitney test. Comparisons with significant differences are denoted in each figure (* = *p* < 0.05, ** = *p* < 0.01, *** = *p* < 0.001, and ns = *p* > 0.05). (A) Total number of genome copies per swab is shown for all sites. Each time point is represented by a different color (day 1 = purple, day 7 = green, day 14 = blue, and day 21 = orange). (B) Total number of genome copies per swab is shown for all timepoints. Each site is represented by a different color (axilla = purple, ear = blue, and groin = orange).

For the axilla and ear sites, the median number of CoNS genomes detected was similar between the intervention and control arms at all time points (Figure 9A,B). From the groin swabs, the quantity of CoNS genomes was significantly higher in the intervention arm (420 genomes per swab) compared to the control arm (125 genomes per swab) (*p*≤ 0.01) on day 7 of sampling (Figure 9C). However, there was no significant difference between the intervention and control arms on day 14 and day 21 of sampling. 

When all sites were combined, the number of CoNS genomes per swab increased from day 1 to day 7 for both the control and intervention arms (*p* ≤ 0.001) (Figure 9A). On day 7, the number of CoNS genomes detected at all sites combined was higher in the intervention arm (median number of genomes = 395) compared to the control arm (median number of genomes = 115) (*p* ≤ 0.001). A similar trend was observed on days 14 and 21 but was not statistically significant (Figure 9A). When all time points were combined and the total number of CoNS genomes was analyzed by site, the median number of detected CoNS genomes per swab was higher in the intervention arm at all sites compared to the control arm (axilla (*p* ≤ 0.05) and groin (*p* ≤ 0.01)) (Figure 9B). 

## 3. Discussion

We investigated the effect of topical coconut oil emollient on the skin microbiome in preterm infants born <30 weeks of gestational age. Microbiome data were collected longitudinally from three body sites up to 21 days after birth. Overall, within-sample diversity was highest on day 1 after birth, with a subsequent decline and emergence of the *Staphylococcus* genus as the dominant taxon from day 7 onwards, consistent with previous studies [16]. qPCR data showed that this increase was largely due to commensal CoNS, rather than *S. aureus.* The coconut oil intervention was associated with lower bacterial diversity within samples and with an overall increase in the density of colonization with CoNS.

The reduction in diversity with age is likely due to a combination of host and environmental factors [16]. CoNS secrete products, such as lipoteichoic acid and proteases, that enhance skin barrier function and immunity [22], limiting colonization with *S. aureus* and other pathogenic microbes. Hence, the increased abundance of the *Staphylococcus* genus may play an important role in the development of the skin’s immune function and compete with other microbes. Moreover, the age-related changes in the skin microbiome may also relate to the neonatal-intensive-care-unit (NICU) environment, which is common to all enrolled infants [22]. Previous studies indicate increasing similarity in NICU environmental and infant skin microbiome with duration of NICU admission over four weeks. Younge et al. [8] reported that *Escherichia*, *Staphylococcus*, and *Streptococcus* genera dominated the environment of preterm infants, taxa that were common on the skin of infants in our study.

Alpha diversity of the skin microbiome was significantly lower in the intervention arm than in the control arm, perhaps related to the higher absolute abundance of CoNS observed in children receiving coconut oil. In contrast, coconut oil application had no effect on the prevalence and absolute quantity of *S. aureus* on the skin of these infants. Indeed, *S. aureus* was detected infrequently and transiently in this study, with only seven infants having detectable levels of *S. aureus* from any site. A recent clinical trial on infants aged 3 to 6 months old found that the use of a glycerol-based emollient increased the alpha diversity of the skin microbiome compared to infants who did not use emollient [23]. Similar studies investigating emollients for the prevention and treatment of other diseases in infants have found that the skin-microbiome alpha diversity is significantly higher in the intervention group compared to the control group [24,25]. However, these differences between studies could be due to differences in the cohorts, as the physical and physiological properties of preterm-infant skin in our study are different from the skin of term infants [11]. Additionally, the antimicrobial effects of coconut oil may have impacted the skin microbiome of the infants in our study, resulting in reduced diversity [26].

We have previously reported that the implementation of topical coconut oil skin care in our NICU was associated with a lower frequency of LOS, without a change in the pattern of causative organisms [20]. The reduction in LOS due to coconut oil may be related to a key component of coconut oil, monolaurin. We have previously demonstrated that, in preterm infants, topical coconut oil administration resulted in higher plasma monolaurin levels compared to skin care without coconut oil [27], with potential direct antimicrobial and immunomodulatory effects. Monolaurin inhibits toxin production and biofilm formation in several bacteria, including *S. aureus* [28,29]. Previously, *S. aureus* has been reported to be inhibited by monolaurin in coconut oil [30].

Antibiotics decrease skin-microbiome alpha diversity in preterm infants [10]; however, in this study, systemic antibiotic treatment had little effect on the skin microbiome. This may be due to insufficient sample size and the almost universal administration of antibiotics in the first few days after birth (94.4%) and a substantial proportion (26.4%) of infants during the following weeks due to suspected sepsis. There have been conflicting reports of the impact of antibiotics on the skin microbiome, and the impact seems to be related to the timing and duration of treatment [8,10,14]. Salava et al. [14] showed that prophylactic benzylpenicillin and netilmicin administered intravenously in the first 5 days after birth significantly decreased the skin-microbial diversity, but no change in skin-microbial diversity was seen when vancomycin and netilmicin were administered intravenously to septic neonates. Another study [10] reported that bacterial richness and diversity were negatively correlated with exposure to intravenous antibiotics for more than 48 h.

Strengths of this study include its prospective integration into a randomized clinical trial comparing preterm infants’ skin care with and without topical coconut oil, including serial sampling of three body sites. However, this study was underpowered to make conclusive findings about associations between the microbiome and antibiotic administration and clinical outcomes such as LOS. This study highlights the rapid succession of the skin microbiome, especially during the first week of life, and the potential modifying effects of topical coconut oil therapy.

## 4. Materials and Methods

### 4.1. Participant Recruitment and Sample Collection

This trial, conducted in the NICU at Edward Memorial Hospital, Perth, was approved by the institutional human research ethics committee (HREC2015191EW) and registered with the Australian Clinical Trial Registry (ACTRN12616000042448). Infants born <30 weeks’ gestational age without major congenital malformations or congenital skin conditions were enrolled. Virgin coconut oil (Nature Pacific, Varsity Lakes, QLD, Australia) was provided in individually sealed 5 mL sachets for each application. Coconut oil (5 mL/kg) was applied by trained nursing staff to neonates in the intervention arm every 12 h for 21 days, commencing within 24 h of birth. The control arm was given standard neonatal care as per NICU guidelines without topical coconut oil [19].

Skin swabs (eNAT FLOQ Swabs^®^, Copan Diagnostics, CA, USA) were collected from the ear lobe, axilla, and groin at 1, 7, 14, and 21 days after birth. Swabs were stored in eNAT tubes, containing a nucleic acid preservation solution, at −80 °C until further processing. Clinical data were extracted from routine electronic databases.

### 4.2. DNA Extraction, PCR, and Amplicon Sequencing

DNA was extracted from the eNAT tubes using the Qiagen Blood Mini Kit with several modifications. Briefly, 1 mL of sample was aliquoted in sterile 2 mL tubes containing 1.0 mm and 0.1 mm sized silicon beads. The tube was centrifuged at 10,000× *g* for 10 min, and 750 µL of supernatant was discarded. A total of 200 µL of AL buffer was added to the sample pellet and resuspended. To this, 20 µL of Proteinase K was added. Sample tubes were placed in Fastprep^®^ (24 Classic Instrument)(MP Biomedicals™, Irvine, USA) for 1 min at a frequency of 4 m/s, after which the samples were immediately incubated for 10 min at 60 °C. Sample tubes were then centrifuged at 8000× *g* for 30 s. A total of 200 µL of chilled 99% ethanol was added, and the tubes were gently mixed by tapping onto the vortex three times at medium speed. A total of 700 µL of supernatant was discarded, and the remaining volume was then used as per the manufacturer’s protocol. DNA was eluted in 20 µL (pre-warmed at 60 °C) of nuclease-free water. The quality of the DNA was analyzed using the NanoDrop 1000 Spectrophotometer, and 4 µL of DNA was used to run 1% (*w*/*v*) agarose gel. DNA was quantified using the Qubit HS dsDNA kit (Invitrogen, Melbourne, Australia). All extracted DNA was stored at −20 °C until further processing.

The 16S rRNA gene V4 region was amplified using 515F and 806R primers with 50 ng of DNA in final volume of 50 µL. Several negative controls were amplified using water as a template. The amplification and amplicon size were confirmed by running samples on 1% (*w*/*v*) agarose gel. The concentration was determined using the Qubit HS dsDNA kit (Invitrogen, Australia). The barcoded amplicons were subsequently pooled at a concentration of 2 ng/µL. The pool was purified using AMPure XP (Beckman Coulter, Australia), and the quality and size of the pool were checked by visualizing on a 1% (*w*/*v*) agarose gel. The composite mixture was sequenced on the Illumina Miseq platform at the UNSW Ramaciotti sequencing facility, Australia.

### 4.3. Bioinformatics and Statistical Analysis

Demultiplexed sequences were quality filtered using QIIME (Quantitative Insights into Microbial Ecology) version 2.2020.2 [31]. Briefly, adapters were removed from paired-end reads using Cutadapt [31]. Demultiplexed sequences were denoised (cutoff 240 bp), filtered, and trimmed with the DADA2 plugin [32]. The sequences were truncated at the base and chimeras were removed. A feature table containing amplicon sequence variants (ASVs) was generated. Finally, the ASVs were classified using the q2-feature-classifier with the SILVA v.138 database self-trained for the V4 region of the 16S rRNA gene.

To remove contaminants that were seen in sequenced controls, we used an in silico approach using the Decontam package in R software (v 4.3.2) [21]. Decontam implements a statistical classification procedure that identifies contaminants that appear at higher frequencies in low-concentration samples and negative controls. All ASVs identified as contaminants were removed from the feature table. The resulting clean feature table was used for all downstream analyses. A de novo phylogenetic tree was generated using log_10_ read counts and the phylogeny align-to-tree-mafft-fasttree plugin for downstream analysis.

All univariate statistical analysis was carried out in the R software using the Vegan package version 2.5.7 [33] and Microbiome package [34]. Alpha (α)-diversity at the ASV level was calculated using the richness, evenness, and Shannon–Weiner diversity index. To compare α-diversity measures between arms, we applied linear mixed models. Random effect models were used to assess longitudinal data, using sample ID as a random effect, to account for multiple samples per participant. We used linear mixed effects models to assess whether coconut oil treatment influenced the skin-microbiome diversity. We fitted a LMM (estimated using REML and nloptwrap optimizer). The normality of the data was confirmed using the Shapiro–Wilk test. For indices that were not normally distributed, such as Chao1 index, we applied a generalized linear model. Beta diversity (β) of microbial communities was calculated with nonmetric multidimensional scaling (NMDS) using Bray–Curtis dissimilarity [35] at the ASV level, and statistical significance was assessed with pairwise ANOSIM. All plots were produced using ‘ggplot2’ [36]. Longitudinal dynamics were visualized using an alluvial plot created with an in-house script for the matplotlib python package. The alluvial plots visualizations were made with the seaborn python package. Differential abundance analysis was carried out in the MaAsLin 2 package [37] in R software v 4.3.2 using random and fixed effect functions with sample ID as a random effect. The *p*-values derived from MaAsLin2 were adjusted using the Benjamin-Hochberg false discovery rate, and q-Values and coefficients were determined. Finally, we created the composition plots using the Fantaxtic v2.0.1 package in R software. In the composition plots, the relative abundance of each taxon is shown at the genus level. Genera with a relative abundance of <10% were listed as “other”.

### 4.4. Quantification and Detection of Staphylococcus *spp.*

*Staphylococcus aureus* and coagulase-negative staphylococci (CoNS) were detected and quantified from extracted DNA using real-time PCR targeting the *spa* (encoding Staphylococcal protein A) and *tuf* (encoding elongation factor Tu) genes respectively. The primers and probe for *spa* were specific for *S. aureus* only, while the primers and probe for *tuf* were specific to all *Staphylococcus* species (Supplementary Table S3). Real-time PCR for the detection of *mecA* (encoding methicillin resistance) and *pvl* (encoding the virulence factor Panton-Valentine leukocidin) was also used (Supplementary Table S3). Reactions were performed in quadraplex, with a final reaction mixture of 1X Taqman FAST Advanced Master Mix (Life Technologies), 0.3 μM of each primer, 0.15 μM of each probe (except for *spa* which had a final concentration of 0.2 μM), 5 μL of template DNA, and nuclease-free water (Integrated DNA Technologies) to a final volume of 20 μL. PCR cycling conditions consisted of an initial denaturation of 95 °C for 2 min, followed by 40 cycles of 95 °C for 30 s and 60 °C for 30 s (data acquiring). All reactions were performed on the QuantStudio™ 6, and data were analysed using QuantStudio Real-Time PCR Software v1.3 (Life Technologies). The quantity of DNA in ng for CoNS and *S. aureus* was determined using a standard curve and an internal positive control. This was converted to total genomes per swab and plotted on a scatter plot. The total amount of CoNS genomes was calculated by subtracting the total number of *S. aureus* genomes if *S. aureus* was detected in that sample. Within each body site, the treatment and control arms were compared using a Mann–Whitney test. Comparison was also made between age of sampling (days 1, 7, 14, and 21).

### 4.5. Quantification and Detection of Bifidobacterium *spp.*

As per the NICU standard protocol at KEMH, all infants received oral supplementation with *Bifidobacterium breve* M16V, independent of skin care allocation. *Bifidobacterium* spp. were detected and quantified from extracted DNA using genus-specific real-time PCR targeting the 16S rRNA gene sequence [38]. Reactions were performed as a single reaction, with a final reaction mixture of 1X Taqman FAST Advanced Master Mix (Life Technologies), 0.3 μM of each primer, 0.15 μM of each probe 5 μL of template DNA, and nuclease-free water (Integrated DNA Technologies) to a final volume of 20 μL. PCR cycling conditions and analysis was as for *Staphylococcal* spp. All sampling ages were compared using a Kruskal–Wallis analysis of variance with Dunn’s multiple comparisons test.

## Figures and Tables

**Table 1:** Demographic characteristics of very preterm infants in control and intervention arms *.

Characteristics	Control Arm (n = 28)	Intervention Arm (n = 28)
Gestation (weeks)	28 (23.7–29.5)	27.6 (24.1–29.3)
Birth weight (g)	984 (424–1534)	1040 (590–1420)
Caesarean delivery	18	15
Male	13	19

* Data are expressed in medians.

**Table 2:** Bacterial genera with relative abundances that differed between body sites or with age at sampling. Differential abundance testing was performed with a mixed effects model in MaAsLin2, using Sample ID as a random effect and body site and age at sampling as fixed effects.

Taxon	Reference	Condition	Coefficient	*q*-Value
*Corynebacterium*	Axilla	Groin	−2.31	0.0001
*Clostridium*	Axilla	Groin	−1.84	9.46 × 10^−5^
*Streptococcus*	Axilla	Groin	1.77	0.002
*Staphylococcus*	Day 1	Day 7	1.68	2.43 × 10^−9^
*Staphylococcus*	Day 1	Day 14	1.71	2.18 × 10^−9^
*Bifidobacterium*	Day 1	Day 7	1.88	0.006
*Cutibacterium*	Day 1	Day 14	−1.88	0.0003

## Data Availability

Data are unavailable due to privacy and ethical restrictions.

## Supplementary Materials

The following supporting information can be downloaded at https://www.mdpi.com/article/10.3390/ijms242316626/s1. (References [39,40] are contained in supplementary material).
