# Supplementary material for: Skin-Microbiome Assembly in Preterm Infants during the First Three Weeks of Life and Impact of Topical Coconut Oil Application
Source: Int J Mol Sci. 2023 Nov 22;24(23):16626. doi: 10.3390/ijms242316626 (PMC10706365; doi:10.3390/ijms242316626)

Supplementary data

Supplementary Table S1. Table of contaminants found in the dataset using *Decontam* software. Contaminants marked TRUE using DNA concentration data. All potential contaminants were removed from the dataset.

Taxon	P-value
D_0__Bacteria;D_1__Proteobacteria;D_2__Gammaproteobacteria;D_3__Enterobacteriales;D_4__Enterobacteriaceae;D_5__Enterobacter	0.002
D_0__Bacteria;D_1__Verrucomicrobia;D_2__Verrucomicrobiae;D_3__Verrucomicrobiales;D_4__Verrucomicrobiaceae;D_5__Prosthecobacter	0.01
D_0__Bacteria	0.09
D_0__Bacteria;D_1__Firmicutes;D_2__Clostridia;D_3__Clostridiales;D_4__Lachnospiraceae;D_5__[Eubacterium] eligens group	0.09
D_0__Bacteria;D_1__Proteobacteria;D_2__Gammaproteobacteria;D_3__Betaproteobacteriales;D_4__Neisseriaceae;D_5__Neisseria	0.03
D_0__Bacteria;D_1__Actinobacteria;D_2__Actinobacteria;D_3__Corynebacteriales;D_4__Corynebacteriaceae;D_5__Corynebacterium 1;D_6__Corynebacterium kroppenstedtii	0.02
D_0__Bacteria;D_1__Firmicutes;D_2__Bacilli;D_3__Bacillales;D_4__Family XI;D_5__Gemella	0.07
D_0__Bacteria	0.05
D_0__Bacteria;D_1__Proteobacteria;D_2__Gammaproteobacteria;D_3__Enterobacteriales;D_4__Enterobacteriaceae	0.06
D_0__Bacteria;D_1__Actinobacteria;D_2__Coriobacteriia;D_3__Coriobacteriales;D_4__Atopobiaceae;D_5__Atopobium;Ambiguous_taxa	0.08
D_0__Bacteria;D_1__Proteobacteria;D_2__Alphaproteobacteria;D_3__Sphingomonadales;D_4__Sphingomonadaceae;D_5__Sphingomonas	0.06
D_0__Bacteria	0.06
D_0__Bacteria	0.08
D_0__Bacteria;D_1__Actinobacteria;D_2__Actinobacteria;D_3__Bifidobacteriales;D_4__Bifidobacteriaceae;D_5__Gardnerella;Ambiguous_taxa	0.09
D_0__Bacteria;D_1__Bacteroidetes;D_2__Bacteroidia;D_3__Bacteroidales;D_4__Bacteroidaceae;D_5__Bacteroides	0.07
D_0__Bacteria;D_1__Firmicutes;D_2__Bacilli;D_3__Bacillales;D_4__Staphylococcaceae;D_5__Staphylococcus	0.08
D_0__Bacteria	0.08
D_0__Bacteria;D_1__Actinobacteria;D_2__Actinobacteria;D_3__Propionibacteriales;D_4__Nocardioidaceae;D_5__Nocardioides	0.03
D_0__Bacteria	0.08
D_0__Bacteria;D_1__Firmicutes;D_2__Bacilli;D_3__Bacillales;D_4__Staphylococcaceae;D_5__Staphylococcus	0.07
D_0__Bacteria;D_1__Actinobacteria;D_2__Actinobacteria;D_3__Propionibacteriales;D_4__Propionibacteriaceae;D_5__Cutibacterium;D_6__[Propionibacterium] humerusii P08	0.05
D_0__Bacteria;D_1__Bacteroidetes;D_2__Bacteroidia;D_3__Sphingobacteriales;D_4__NS11-12 marine group;Ambiguous_taxa;Ambiguous_taxa	0.04
D_0__Bacteria	0.02
D_0__Bacteria;D_1__Firmicutes;D_2__Clostridia;D_3__Clostridiales;D_4__Lachnospiraceae;D_5__Blautia	0.01
D_0__Bacteria;D_1__Proteobacteria;D_2__Gammaproteobacteria;D_3__Betaproteobacteriales;D_4__Burkholderiaceae;D_5__Tepidimonas	0.01
D_0__Bacteria;D_1__Firmicutes;D_2__Bacilli;D_3__Bacillales;D_4__Staphylococcaceae;D_5__Staphylococcus	0.01
D_0__Bacteria;D_1__Actinobacteria;D_2__Actinobacteria;D_3__Corynebacteriales;D_4__Corynebacteriaceae;D_5__Corynebacterium 1;D_6__Corynebacterium sp. NML98-0116	0.04
D_0__Bacteria;D_1__Firmicutes;D_2__Bacilli;D_3__Bacillales;D_4__Staphylococcaceae;D_5__Staphylococcus	0.01

D_0__Bacteria	0.07
D_0__Bacteria;D_1__Firmicutes;D_2__Bacilli;D_3__Bacillales;D_4__Staphylococcaceae;D_5__Staphylococcus	0.09
D_0__Bacteria	0.07
D_0__Bacteria;D_1__Firmicutes;D_2__Bacilli;D_3__Bacillales;D_4__Staphylococcaceae;D_5__Staphylococcus	0.02
D_0__Bacteria;D_1__Patescibacteria;D_2__Parcubacteria;D_3__Candidatus Nomurabacteria;D_4__uncultured bacterium;D_5__uncultured bacterium;D_6__uncultured bacterium	0.02
D_0__Bacteria;D_1__Firmicutes;D_2__Bacilli;D_3__Bacillales;D_4__Bacillaceae;D_5__Anaerobacillus;D_6__uncultured bacterium	0.07
Unassigned	0.08
D_0__Bacteria;D_1__Bacteroidetes;D_2__Bacteroidia;D_3__Bacteroidales;D_4__Prevotellaceae; D_5__Prevotella;D_6__Prevotella bivia	0.01
D_0__Bacteria;D_1__Epsilonbacteraeota;D_2__Campylobacteria;D_3__Campylobacterales;D_4__Campylobacteraceae;D_5__Campylobacter;D_6__Campylobacter ureolyticus	0.06
D_0__Bacteria;D_1__Firmicutes;D_2__Bacilli;D_3__Bacillales;D_4__Staphylococcaceae;D_5__Staphylococcus	0.08
D_0__Bacteria;D_1__Firmicutes;D_2__Bacilli;D_3__Bacillales;D_4__Staphylococcaceae;D_5__Staphylococcus	0.09
D_0__Bacteria;D_1__Firmicutes;D_2__Clostridia;D_3__Clostridiales;D_4__Clostridiaceae 1;D_5__Clostridium sensu stricto 1	0.04

Supplementary Table S2. Number of *Staphylococcus aureus* genomes detected per swab by qPCR. Samples from which no *S. aureus* DNA was detected are not shown. The primers/probes for the *spa* genes were used for the detection of *S. aureus.*

**Participant ID**	**Site**	**Time**	**Intervention/control**	**Genomes per swab**
103	Groin	Day 14	Control	771.5176887
103	Groin	Day 21	Control	173.8599047
120	Groin	Day 21	Control	187.1580689
127	Groin	Day 7	Intervention	44.28708505
128	Groin	Day 21	Control	43.75260674
129	Groin	Day 7	Intervention	170.1575791
210	Groin	Day 21	Control	81.95188919
112	Ear	Day 14	Control	685.0316174

Supplementary Table S3. Primers and probes used for absolute quantification of *Staphylococcus* and *Bi idobacterium* species in qPCR.

Species	Primer/probe	Sequence (5' - 3')	Target	Reference
**CoNS**	Forward	TATCCACGAAACTTCTAAAACAACTGTTACT	tuf	Okolie et al., 2015 (39)
	Reverse	TCTTTAGATAATACGTATACTTCAGCTTTGAATTT		
	Probe	TATTAGACTACGCTGAAGCTGGTGACAACAT		
S. aureus	Forward	AAGATGATCCAAGCCAAAGTGCTAA	spa	This study
	Reverse	TCATAGAAGGCGCTTTGTTGATCTT		
	Probe	GGTGAAGCTCAAAAACTTAATGACTCTC		
S. aureus	Forward	TTACACAGTTAAATATGAAGTGAACTGGA	pvl	Adapted from Okolie et al., 2015 (39)
	Reverse	AGCAAAAGCAATGCAATTGATG		
	Probe	ACTCATGAAATTAAAGTGAAAGGACA		
S. aureus	Forward	TGGTATGTGGAAGTTAGATTGGGAT	mecA	Nakagawa et al.. 2005 (40)
	Reverse	CTAATCTCATATGTGTTCCTGTATTGGC		
	Probe	TTCCAGGAATGCAGAAAGACCAAAGCA		
Bifidobacterium spp.	Forward	GCGTGCTTAACACATGCAAGTC	16s rRNA gene	Penders et al., 2005 (38)
	Reverse	CACCCGTTTCCAGGAGCTATT		
	Probe	TCACGCATTACTCACCCGTTCGCC		

Supplementary Figure S1. Alluvial plots of longitudinal microbial profile for axilla, ear and groin sites. The alluvial plots show the microbial dynamics for t01=Day 1, t02=Day 7, t03= Day 14 and t04= Day 21. Each participant is represented as a line, coloured by the dominant bacterial genus at that time point.

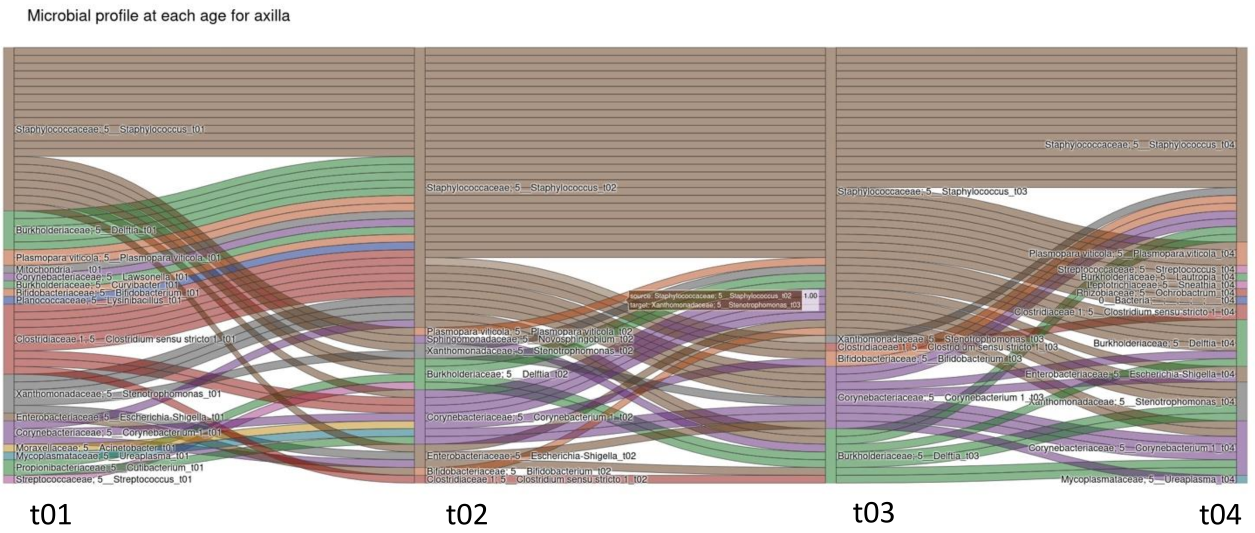

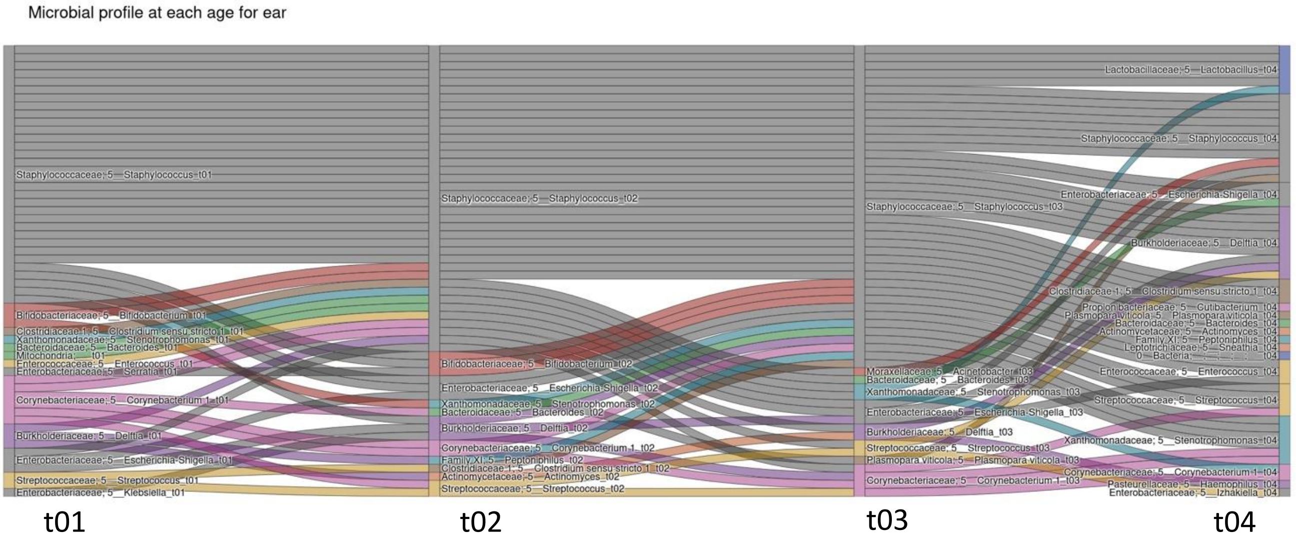

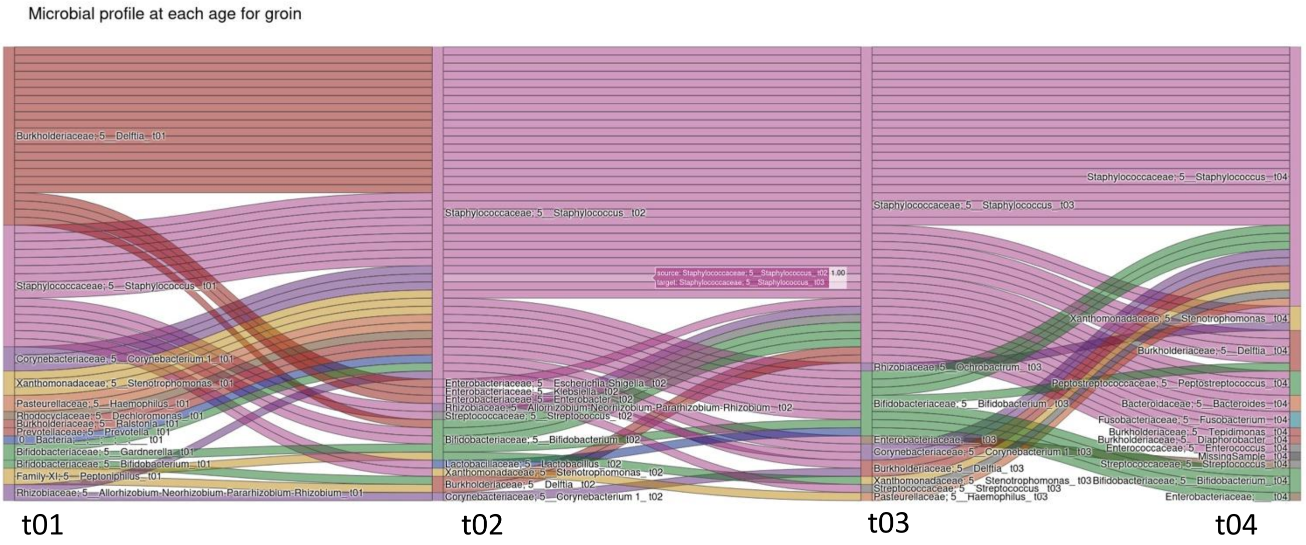

Supplementary Figure S2. NMDS plot for each site in intervention and control group. Beta diversity shown in a Non-metric multidimensional scaling (NMDS) plot using Bray-Curtis dissimilarity indices for each body site in control and intervention groups at ASV level for Day 7 age at sampling.

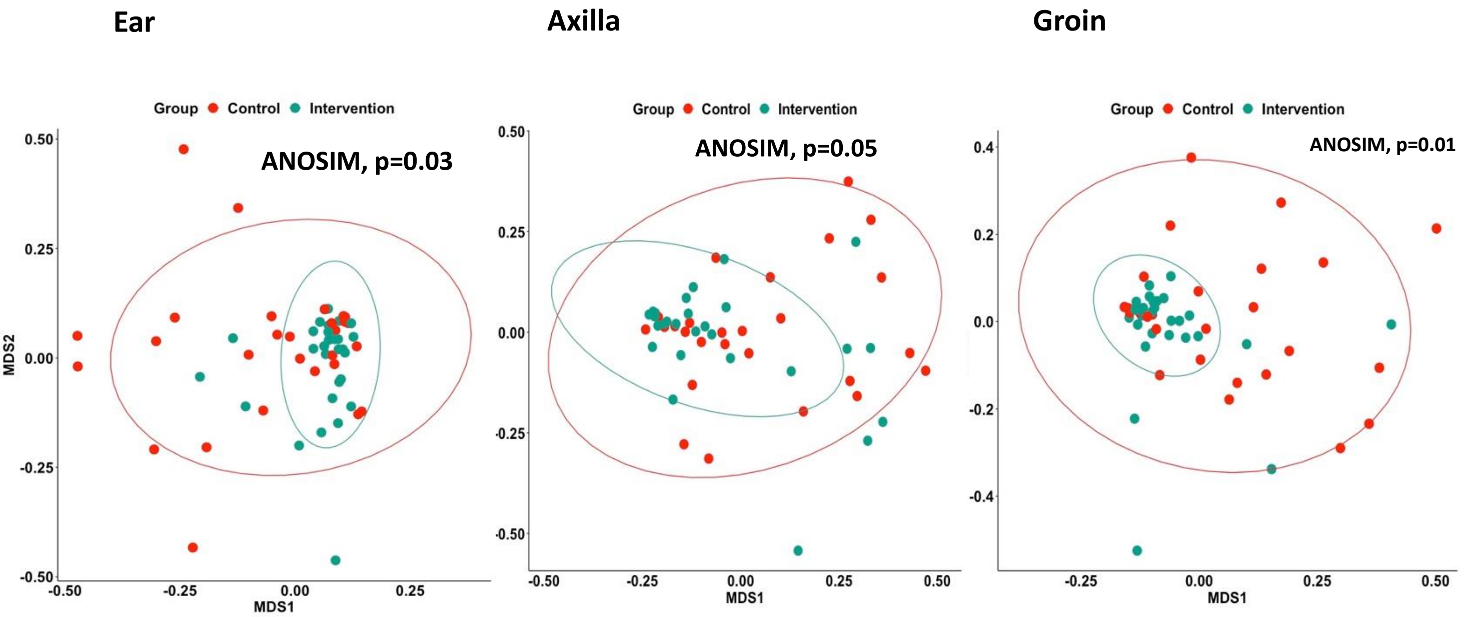

Supplement: Supplementary file 1 [file ijms-24-16626-s001.zip › ijms-2719597-supplementary.pdf]
